# Networked control system with MANET communication and AODV routing

**DOI:** 10.1016/j.heliyon.2022.e11678

**Published:** 2022-11-18

**Authors:** Abhay Bhatia, Anil Kumar, Arpit Jain, Adesh Kumar, Chaman Verma, Zoltan Illes, Ioan Aschilean, Maria Simona Raboaca

**Affiliations:** a Roorkee Institute of Technology, Roorkee, Uttarakhand, India; b Faculty of Engineering & CS, Teerthanker Mahaveer University, 244001 Moradabad, India; c Department of Computer Science & Engineering, Koneru Lakshmaiah Educational Foundation, Vaddeswaram, Andhra Pradesh, 522502, India; d Department of Electrical & Electronics Engineering, School of Engineering, University of Petroleum and Energy Studies, 248001, Dehradun, India; e Department of Media and Educational Informatics, Faculty of Informatics, Eötvös Loránd University, 1053 Budapest, Hungary; f Faculty of Civil Engineering, Civil Engineering and Management Department, Technical University of Cluj Napoca, Cluj-Napoca, Romania; g Faculty of Building Services, Technical University of Cluj Napoca, 400114, Cluj-Napoca, Romania; h Doctoral School, University Politehnica of Bucharest, Splaiul Independentei Street, No. 313, 060042 Bucharest, Romania; i National Research and Development Institute for Cryogenic and Isotopic Technologies-ICSI Rm. Vâlcea, Uzinei Street, No. 4, P.O. Box 7 Râureni, 240050 Rm. Vâlcea, Romania

**Keywords:** AODV, MANET, NCS, NS2, PDR, Throughput, Control overhead, End-to-end delay, Wireless communication

## Abstract

The industries are presently exploring the use of wired and wireless systems for control, automation, and monitoring. The primary benefit of wireless technology is that it reduces the installation cost, in both money and labor terms, as companies already have a significant investment in wiring. The research article presents the work on the analysis of Mobile Ad Hoc Network (MANET) in a wireless real-time communication medium for a Networked Control System (NCS), and determining whether the simulated behavior is significant for a plant or not. The behavior of the MANET is analyzed for Ad-hoc on-demand distance vector routing (AODV) that maintenances communication among 150 nodes for NCS. The simulation is carried out in Network Simulator (NS2) software with different nodes cluster to estimate the network throughput, end-to-end delay, packet delivery ratio (PDR), and control overhead. The benefit of MANET is that it has a fixed topology, which permits flexibility since mobile devices may be used to construct ad-hoc networks anywhere, scalability because more nodes can be added to the network, and minimal operating expenses in that no original infrastructure needs to be developed. AODV routing is a flat routing system that does not require central routing nodes. As the network grows in size, the network can be scaled to meet the network design and configuration requirements. AODV is flexible to support different configurations and topological nodes in dynamic networks because of its versatility. The advantage of such network simulation and routing behavior provides the future direction for the researchers who are working towards the embedded hardware solutions for NCS, as the hardware complexity depends on the delay, throughput, and PDR.

## 1 Introduction

**Figure 1:**
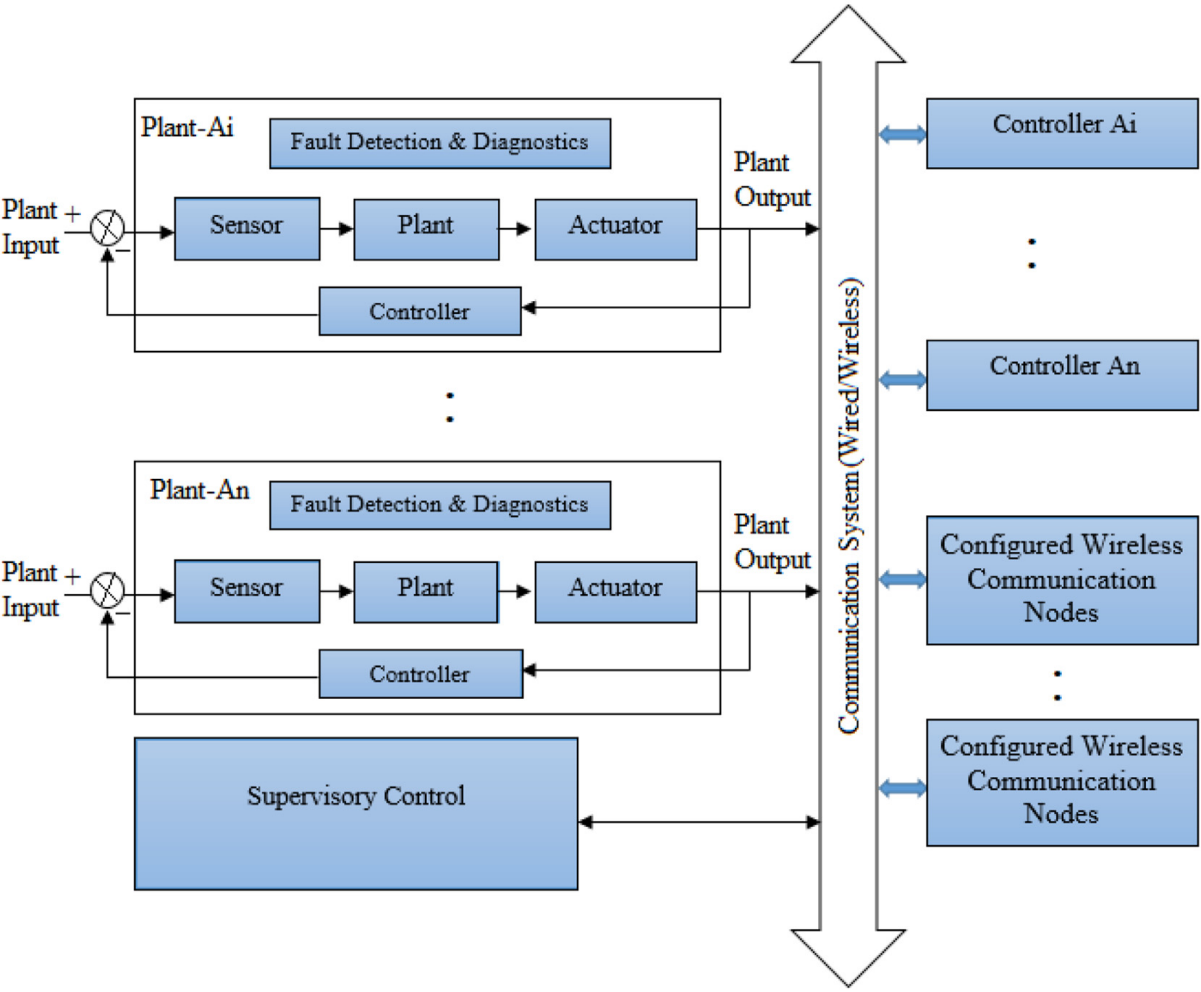
NCS in communication [2].

The NCS is made up of numerous connected devices that communicate with each other through various networks. Industrial automation, building monitoring, and automobile control are examples of NCS. NCS [1] has many benefits including quick deployment, wide dissemination, and flexible, and adaptive system architecture. However, several drawbacks including information loss and data transmission delays could affect system performance and should be considered into account while designing the system. Figure 1 suggests the scheme of bidirectional communication in a plant with multiple embedded sensors. There is one supervisory control system in which different nodes can communicate in specific wired or wireless networks. The MANET [3] nodes in the communication infrastructure can be set up in a particular architecture. Different techniques have been used to reduce the communication infrastructure between sensors and controller nodes while maintaining the stability of the control system. The most essential issue in the NCS fault detection system is the network-induced delay, which includes sensor-to-controller and controller-to-actuator delays. The MANET cannot be used directly in a wireless system because of its resource requirements for sensor nodes, user interface requirements for node devices, processing speed, and node density requirements. A composite routing solution for MANET and wireless networks is necessary to utilize the network infrastructure efficiently for maximum energy and enhance the network's lifespan.

**Figure 2:**
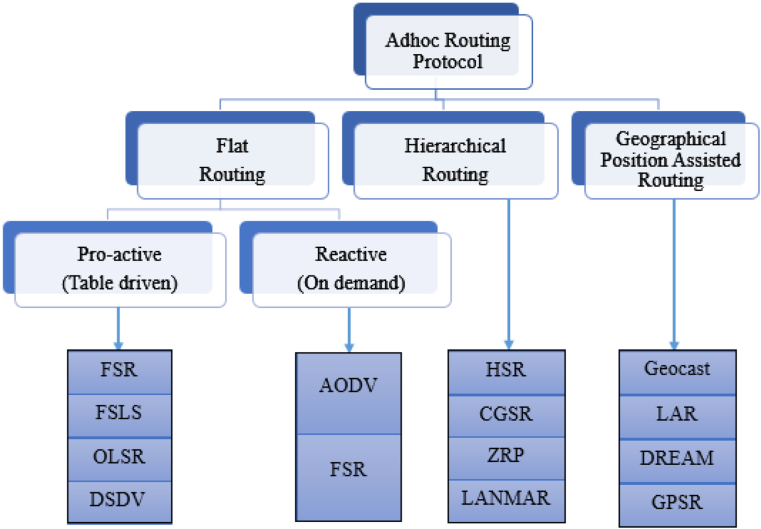
Routing protocols in MANET.

The inclusion of the communication network in a feedback control loop complicates NCS design and analysis because it introduces extra time delays in control loops, and enhances the possibility of packet loss. The motivation for the work is that NCS supports wireless communication networks such as Zigbee, Bluetooth, Zwave, and Wifi. MANET architectures have been realized using WLAN Zigbee (IEEE-802.15.4) nodes that work in the physical radio specification [5] to operate in unlicensed bands including 868 MHz, 900 MHz, and 2.4 GHz with low latency, low duty cycle, secured protocols, and the maximum capacity of 65000 nodes. The Zigbee technology supports star, cluster-tree, and mesh (peer-to-peer topology) to configure the nodes in the simulation environment. There are two types of routing on the MANET: proactive and reactive unicast protocols. A proactive protocol is one that regularly requests updated information from surrounding nodes and shares routing tables to maintain an up-to-date routing table. The reactive protocol requests a route from the source to the destination when a node wants to send a packet but there is not a valid path in the routing table. Figure 2 depicts the classifications of the routing protocols. Flat, hierarchical, and geographical routing are the three basic types of routing protocols. There exist two basic arrangements within flat routing protocols: proactive protocols and reactive protocols [4]. The reactive protocols only seek information about the setup when a packet has to be transmitted, but proactive protocols maintain their topological routing tables by continuously sending out maintenance packets around the setup. To create hierarchical structures on the flat network, hierarchical routing systems [5] typically use clustering techniques, in which each cluster is associated with the master node that is in charge of it and the infrastructure it shares with other clusters.

Geographical routing refers to the use of a node's geographic location to help establish routing paths. This imposes the maintenance of regular information in the geographical locality and present network location. Dynamic source routing (DSR) is a reactive protocol that uses deleting the periodic table-update messages necessary in the table-driven technique to reduce the bandwidth wasted by control packets in wireless networks to provide route discovery [6, 7] and management. The zone routing protocol (ZRP) is a hybrid protocol that employs both reactive and proactive methods, allowing them to complement one another strengths. This protocol has been considered widely to decrease the amount of communication overhead in comparison to pure proactive methods [8]. Additionally, it reduces the delays that are associated with reactive approaches like DSR. The protocol creates a zone around a node, the size of which depends on the hop count ‘p’. The proactive protocol used for communication within the zone is the intra-zone routing protocol (IARP). It is a group of link-state protocols that keep track of routing information for nodes in a specific node's zone. Global state routing (GSR) is a protocol based on the classic link state method. These link state tables assist in reducing the number of control messages [9], sent across the network. The updated message size is slightly larger than the older messages received at the user end because it exchanges links. Fisheye state routing (FSR) is a descendant of GSR, and it adjusts the message size in GSR by updating neighboring nodes. It has a feature of scalability but has a lot of inaccuracy. To overcome this, distant locations receive updates at a frequency inversely correlated with their level of mobility. The wireless routing protocol (WRP) assurances loop autonomy by escaping temporary routing loops by using the successor information. Moreover, WRP necessitates the maintenance of four routing tables on each node. As the network grows in size, routing imposes a large amount of memory cost at each node [10]. It assures that messages are exchanged with a greeting message and that no additional messages are exchanged between the nodes. It also consumes significant energy to keep the node active at all times. The Distance Routing Effect Algorithm for Mobility (DREAM) is a routing technique that follows a different approach to route than other techniques. Each node in DREAM has a GPS and hence knows its geographical coordinates, which are periodically transferred between nodes and saved in the routing table. Broadcasting is commonly used in routing protocols as part of their route discovery and maintenance operations.

The contribution of the work is as follows: The AODV routing simulation is carried out for MANET and NCS with different cluster sizes in NS2 simulation. The performance of the system is estimated using different performance indices such as PDR, throughput, end-to-end latency, and control overhead. The simulation is the preliminary work to understand the behavior of the MANET in NS2 to further extend it on the hardware platform. The work is limited to network simulation only. The rest of the paper is organized as follows. The related work is described in Section 2. NCS modeling is discussed in Section 3. The AODV routing and methodology flow are given in Section 4. The Section 5 presents the simulation results and discussions with various clusters, followed by the conclusion in Section 6.

## 2 Related work

MANET is a self-configuring system of mobile routers associated with wireless links but without the need for a contact point. Each mobile device in the system is self-contained. Mobile gadgets [11] are restricted to moving around and consolidating themselves in whatever way they want. The wireless medium is divided by the MANET nodes, and the system architecture varies irregularly and energetically. The communication links in MANET are regularly damaged, and nodes are free to migrate to any location. The number of nodes and the density of nodes are determined by the real-time usages in which MANET is used. Major network applications that have emerged as a result of MANET include data networks, wireless sensor networks, scheduling networks, and device communication networks. There are still some design flaws and obstacles to address with many applications. The main objective of mobile ad-hoc networks is to expand mobility into the world of autonomous and mobile wireless domains, where the architecture supports a large number of ad-hoc nodes and network routing that can serve both hosts and routers. Several security vulnerabilities [12] as well as several remedies have been identified in wireless systems like MANET. However, very few are capable to guarantee that is unrelated to the critical security challenges. With these factors in mind, the main objective of mobile Adhoc networking is to add routing capabilities into mobile communication nodes to provide steady and proficient functioning in mobile wireless setups. The networks are based on multi-hop topologies, which are expected to be dynamic, random, fast-changing, and support bandwidth-constrained wireless contacts in such networks.

The admission control technique [13] is followed and works with both individual and multipath routing protocols in the WSN control system. The different types of simulation work have been done to find the suggested QoS framework may harmonize flawlessly with multipath routing protocols, resulting in substantial gains incomplete system performance, particularly for challenging applications such as real-time signal, video, and telephony applications. The strategy involves the creation of an energy-efficient layer channeling protocol [14] in the specified network for military applications, as well as simulation using a revolutionary cross-layer design methodology to improve the network's stability and lifespan. The effectiveness of this strategy and simulation model was analyzed using NS2 software. The simulation was done for the optimized link-state routing protocol (OLSR) method [15], which provides the lesser delay than the applied AODV protocol. The OLSR method resulted in a delay of 0.02 s compared to 0.156 s for the AODV protocol, whereas the AODV protocol outperforms OLSR in all other performance measures. PDR is 11% lower, throughput is 8% lower, packet loss is 46% higher, energy consumption is 49% higher, and routing overhead is 94% greater than OLSR. This finding demonstrates that the AODV protocol is more adaptable to changes in topology than the OLSR protocol. Secured and optimized (SO) AODV routing [16] was used for encryption and decryption-based cryptographic wireless data communication on the NS2 simulator to estimate the parameters such as delay, throughput, and PDR. The combined approach of refreshing and load matching [17] was used to explain the problem of fast battery exhaustion and blocking in WSN. The basic strategy of re-charging is used to keep each node in the system at the preferred energy level. The modified AODV implementation enhanced WSN performance based on energy levels, throughput, and PDR. The security methods [18] are primarily essential to safeguard the system from security threats and to secure the network. In reality, cryptography techniques are frequently used to ensure security. Moreover, the technologies are unsuitable due to the sensor node's lack of power and memory that is low computation, and limited energy reserves. As a result, one of the most critical research issues that has been identified in WSN is ensuring security while respecting the sensors' particular limits. The performance of the AODV routing chip is investigated using field-programmable gate array (FPGA) synthesis hardware [19] parameters such as slices, look-up tables (LUTs), input/output blocks (IoB), flip-flops, and memory utilization for various cluster configurations (N = 10, 20,…,100). The latency and frequency are also estimated on the Virtex-5 FPGA. The main problems of the WSN parameters are scalability and routing [20]. Modelsim 10.0 simulation software was used to verify node communication and performance. The FPGA hardware and timing characteristics are investigated for various node cluster configurations (N = 10, 20,…,150). The machine learning prediction is built using the OLSR routing protocol network performance parameters. The WSN routing is also configured using different topologies [21], and 2D and 3D routers [22, 23]. The network design is considered based on mesh, tree, and ring network-on-chip [24, 25] to estimate the hardware chip performance for different configured nodes. The 3D multilayer nodes configuration was also verified on the hardware platform to estimate the delay [26]. In wireless communication, different routing and topology are followed to estimate the network performance for a network control such as smart grid communication based on Zigbee IEEE 802.15.4 using mesh, cluster tree, and ring topological communication [27, 28]. The monitoring and control operations of the power plant control system were also verified using embedded hardware chips [29]to estimate the delay and focused on the security enhancement of the system. A smart grid network connects an electrical distribution system to a network of information and communication. The layered technique is used to design, develop, and build communication network protocols [30]. Each layer is created to perform a certain purpose in conjunction with the others. The work was also carried out in the direction to get the performance of the multifunctional photovoltaic system [31] domestic system and distribution. The multi-region power control system [32] was developed to understand the behavior of primary and secondary voltages. The system is applicable for smart grid and control applications [33]. The geothermal electric submersible control system was investigated based on sensorless control induction and LC filtering [34]. Such systems need communication in real-time and routing to play an important role. The fuzzy-based approach is also proposed by choosing the most trustworthy nodes to design the direction between [35] the source and destination nodes.

The performance of the AODV reactive routing protocol was improved using the properties of the input of the node via a fuzzy implication system to calculate the value of the node expectation level, which can be utilized as a measure to create an ideal route from source nodes to destination. The AODV routing method was proposed without taking into account the link between communication and control, which is incompatible with wireless networked control applications [36]. This investigation makes use of AODV routing, in an IEEE 802.15.4 network that is used to configure the temperature control system. The results presented in the scenarios reveal that AODV routing can choose a data transmission route with a large volume of traffic. Although packet communication errors occur, it takes longer before choosing a new route. As a result of the regular packet transmissions and collisions, the nodes consume more energy, and the intended control objective cannot be contented quickly. MANET has been used in smart cities, cab sharing, intelligent traffic control, ambulance services, integrated mobile cloud, 5G, 6G, and industrial power plants for monitoring. MANET supports different types of services such as air force UAVs, robots, street light networks, army tactical, smartphones, internet-based communication, and disaster rescue.

MANET enables users to interconnect directly and play a vital role in smart communication and IoT applications through ubiquitous smart devices, and embedded sensors in networked control systems. The NCS can create self-configuring MANETs in such smart surroundings to send and receive data packets to a destination over numerous hops via intermediate nodes. When the communication is done in WSN-based NCS, the PDR, delay, throughput, and control overhead play an important role. The performance of the NCS can be estimated using these parameters. The objective of the research work is to estimate these parameters using NS2 simulation for AODV routing. Industries are working towards the cost and power-efficient embedded hardware chip design and integrating with NCS to provide faster switching using field-programmable gate array (FPGA). In the current state of the art, FPGAs are becoming increasingly used in the design of industrial control systems. FPGA-based speed controllers, motor controllers, power plant control, and monitoring are some examples that are already proven for NCS. An FPGA can be reconfigured and reprogramed during system operation via dynamic partial reconfiguration (DPR) making the network more scalable and reliable. The simulation of MANET and AODV routing on NS2 with different cluster nodes, and estimation of delay and throughput provides the direction to the hardware design engineers to pre-estimate the MANET for growing hardware requirements and multicore process chips. As pointed out that the Zigbee WSN supports star, mesh, and cluster tree topology. The MANET protocol measures range and throughput using Zigbee-based communication. The MANET modeling in NS2 will provide a great platform for software and hardware engineers with different perspectives.

## 3 NCS modeling

Networked Control System (NCS) [37] is a closed feedback control system that routines a real-time communications network to transfer information between the controller and plant. The plant sensor perceives the present state of the plant and directs it to the controller with the help of a real-time communication network. The controller equates the present state of the plant [38] to an existing value, computing the accurate control value that the plant requires, and sending it back to the plant actuator. The NCS is made up of a band-limited, bidirectional digital communication network that is substantially and scientifically linked to a spatially disseminated control system [39] that operates on a specific plant. The network transmits digital data such as switch control signals, sensor signals, actuator signals, and operative inputs. The network connects all nodes of the control system, including the computer, directorial network manager, actuators, software, hardware controller, and sensors. The architecture of the common communication system is protected by the feedback control system loops [40].

The NCS has three primary components of the closed-loop: computational operation of the system, communication network for real-time processing, and closed-loop system control. The controller relies on computing system functionality to determine the present state of the plant, compares it to the reference model, and computes the updated value from the controller. The routing protocols, packet drops, and jitter can cause delays in real-time communication networks [41]. NCS is a form of control system that uses a serial communication network to close the control loop. The control community has shown a lot of awareness of such system configurations, which are based on the concept of integrating communication systems and control networks [42]. The same behavior is referred to other names for NCS, including network-based control systems, and control over networks.

NCS is based on a direct and hierarchical structure. A direct structure is made up of a controller and a remote system with a physical plant, actuators, and sensors. To achieve remote closed-loop control, the controller and the plant are physically placed at diverse places and are directly associated using a data communication network.

**Figure 3:**
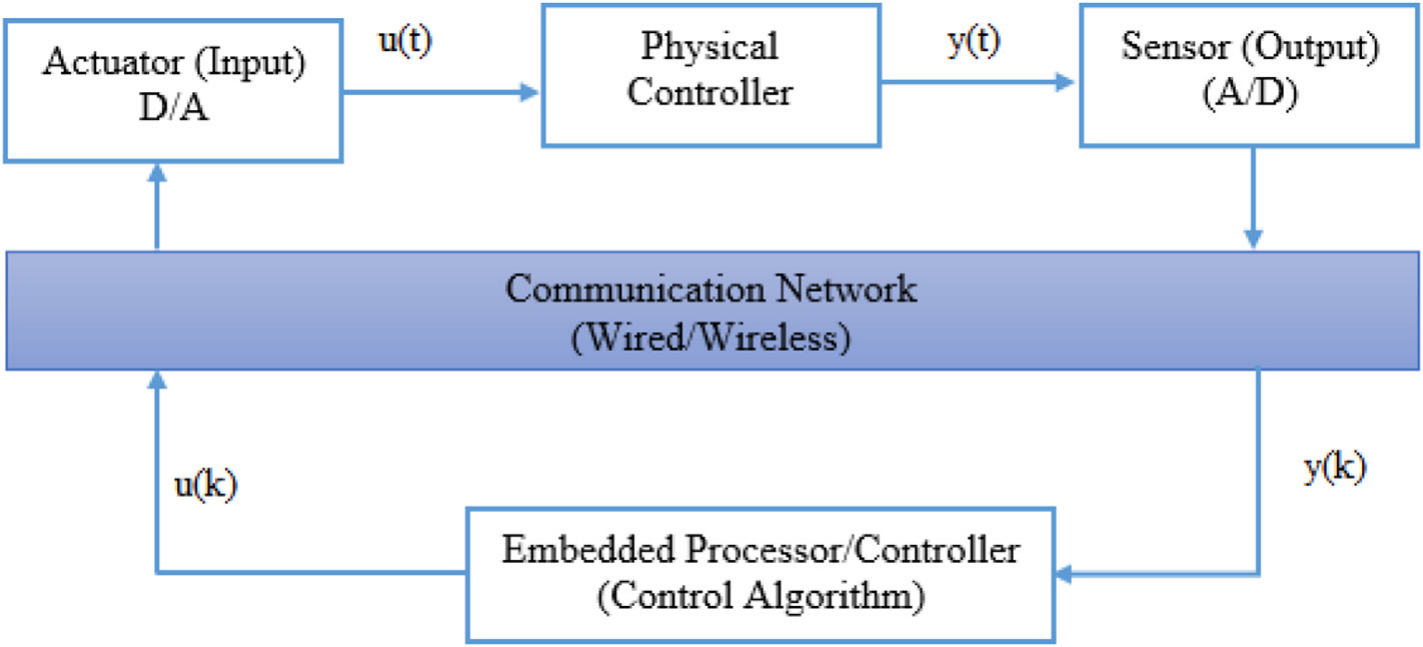
Communication in NCS.

**Figure 4:**
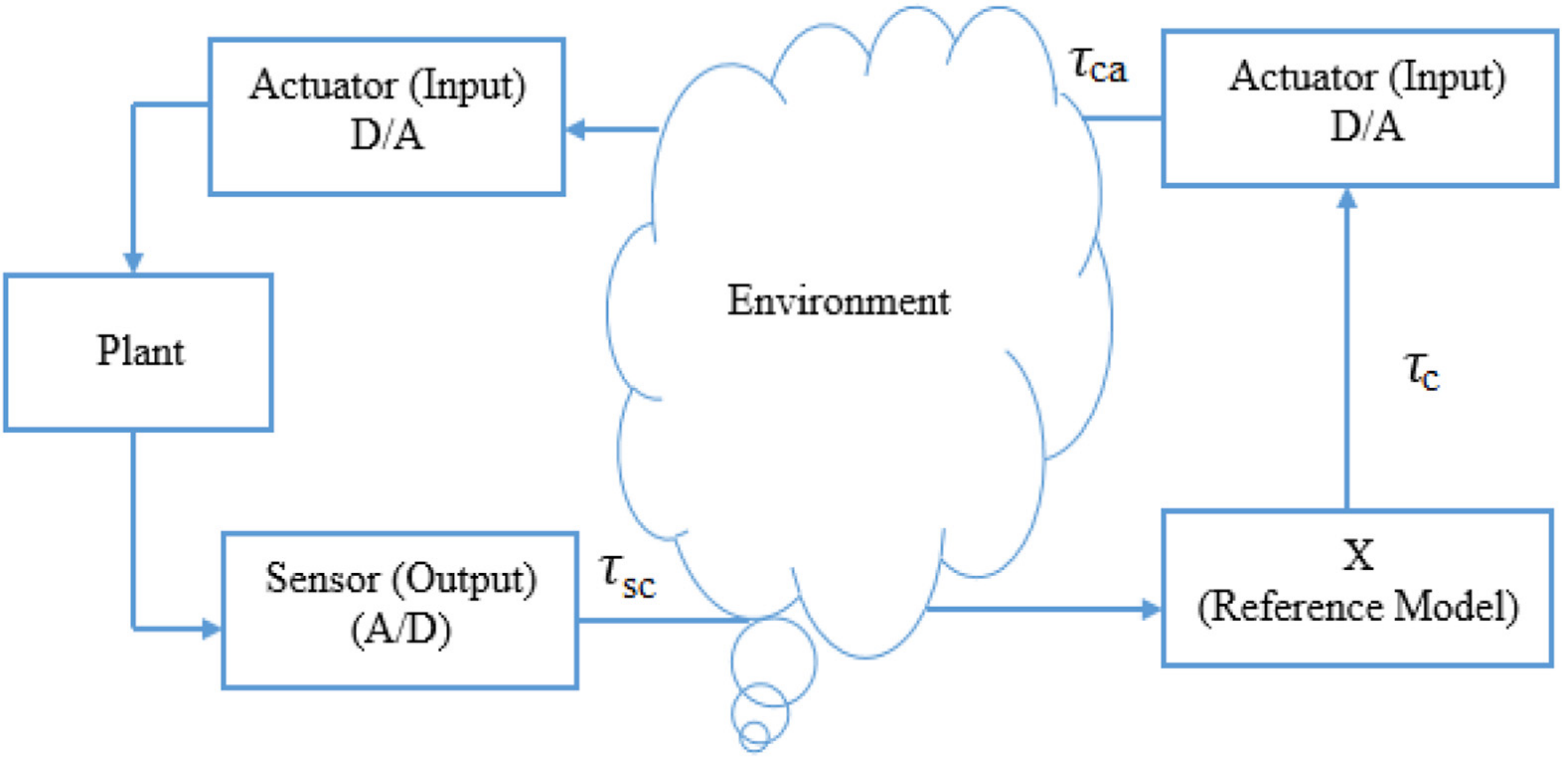
Delay model.

Figure 3 shows a schematic description of the NCS system for direct realization. The usage of a communication network increases delays [43, 44] between the controller and plant, which is a serious issue in NCS. Figure 4 depicts the delay model of the NCS. Eq. (1) presents the overall system delay.

(1) $$\tau_{tot}=\tau_{sc}+\tau_{c}+\tau_{ca}$$

where, τ_sc_ presents the delay that takes for the plant sensor (output) to reach the controller, τ_c_ is the computational delay, τ_ca_ is the delay associated from the controller to the plant, and τ_tot_ is the overall system delay.

The value of the computational delay is very small in plant operation and thus it can be neglected. The equation of the total delay can be rewritten as Eq. (2),

(2) $$\tau_{tot}=\tau_{sc}+\tau_{ca}$$

Wireless sensor networks (WSN) [45] have attracted a lot of attention as a novel communication paradigm for industrial cyber-physical systems. The router is an essential element of industrial WSN due to its implications for the reliability, latency, and schedulability of wireless communication. MANET nodes do not know their network topology. Despite that, they must learn it from themselves because the topology in an ad-hoc network is dynamic. MANET routing protocols must adapt to deviations in the network topology, and keep routing information so that the desired packets can be routed to their destinations. Because of the active topological architecture and characteristics of MANETs, security is far more difficult than in typical network settings filled with the primary controller. The fundamental requirement for the new node is that it must broadcast its presence and arrival whenever it joins an ad hoc network and wait for similar announcement broadcasts from existing mobile nodes. In the wireless channel, the average end-to-end delay is used. The total end-to-end delay of the packets is calculated using Eq. (3)

(3) $$Delay\;\left(t_{av}\right)=\frac{\sum_{1}^{n}\left(t_{pd}-t_{ps}\right)\;}{\sum_{1}^{n}N_{r}}$$

The PDR is referred to the ratio of the number of packets received by the destination node to the number of packets sent from the source. Eq. (4) presents the relation of PDR.

(4) $$PDR=\frac{N_{r}}{N_{s}}$$

Throughput is determined by multiplication in the numbers of the packet transmitted and the size of the packet per unit of time. The average throughput is considered in bits per second (bps) and presented using Eq. (5).

(5) $$Throughput\;\left(Th_{av}\right)=\frac{N_{r}\times {\left(Size\right)}_{d}}{T_{s}\times t_{av}}$$

Control overhead (CH) is the proportion of control packets supplied to real data received at each MANET node.

CH = Control packets supplied/ real data received

where, $t_{pd}$ is the transmission time $t_{ps}$ is the generation time at the source $N_{r}$ are the number of packets received by the destination $N_{s}$ are the number of packets transmitted from the source $T_{s}$ is the time of simulation ${\left(Size\right)}_{d}$ is the packet size in the simulation environment which is not constant in the simulation.

## 4 AODV routing & methodology

AODV routing [46] has the advantage that it does not require additional overhead against data packet transmissions, as it does not require the use of source routing. The route error (RERR) message is delivered through a node detecting the link interruption. If the connection is lost and the path is no longer functional, then the messages cannot be sent. In this case, other nodes will recast the message. The RERR notification displays the unreachable location. The route is deactivated by message-receiving nodes. The behavior of the AODV routing supports unicast and multicast simulation environments even if the nodes are in constant movement. AODV routing is reliable, loop-free, and does not require a centralized wireless mesh network system to conduct the wireless network routing procedure. The time for setting and releasing the connections to determine the destination and last route is eliminated in AODV routing, as it is self-starting and loop-free.

The AODV protocol provides on-demand routing to destinations and supports both unicast and multicast routing [47]. The protocol only generates the path between nodes if the source nodes send the request to them. As a result, AODV is considered an on-demand technique because it generates no additional network traffic. The links are continued for a long duration based on the source node requirements. They also create links to connect the users in the multicast environment. To guarantee route clearness, the AODV services order numbers that are self-maintaining and loop-free, and they can engage more mobile users. The links in AODV persist silently in anticipation of connections associated with the system. Different connection requests are disseminated by network nodes in the necessity of links. The message is forwarded by the other AODV nodes, who keep note of which node issued the connection request. As a result, a sequence of momentary routes is formed back to the requesting node. A node delivers a progressive message to the asking node via temporary routes when it receives such messages and has a path to the preferred node. The node request is processed through the path with the minimum hops across other nodes. The directed tables gradually recycle any accesses that are not used. The sending node receives the routing error in case of link failure and the process is repeated.

**Figure 5:**
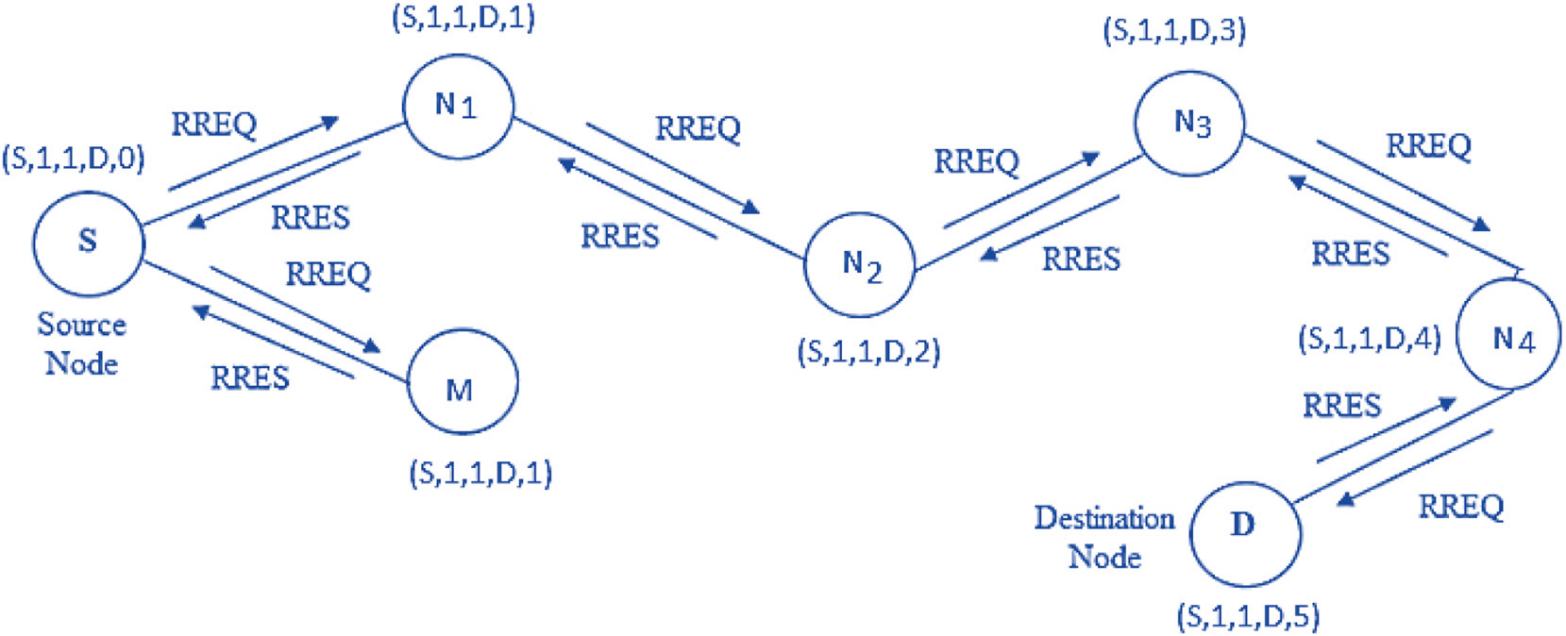
AODV routing example.

Consider a network with seven nodes labeled as ‘S’, ‘N_0_’, ‘N_1_’, ‘N_2_’, ‘N_3_’, ‘N_4_’, and ‘D’, as denoted in Figure 5. The source node (S) and destination node (D) are linked with some intermediate paths. Two things must be maintained in AODV routing, one is the sequence number and the other is the broadcast identification document (ID). The destination ID information is already available. As a result, keeping track of the destination order and updating the route from the source to the destination is simple. The destination ID information is already available. As a result, the possession trail of the destination series number and updating the path from source to destination is simple. Route request (RREQ), and route response (RREP) are two important signals that are used to convey an application from the source to the destination for route discovery and a response from the destination to the source against the expected response.

**Figure 6:**
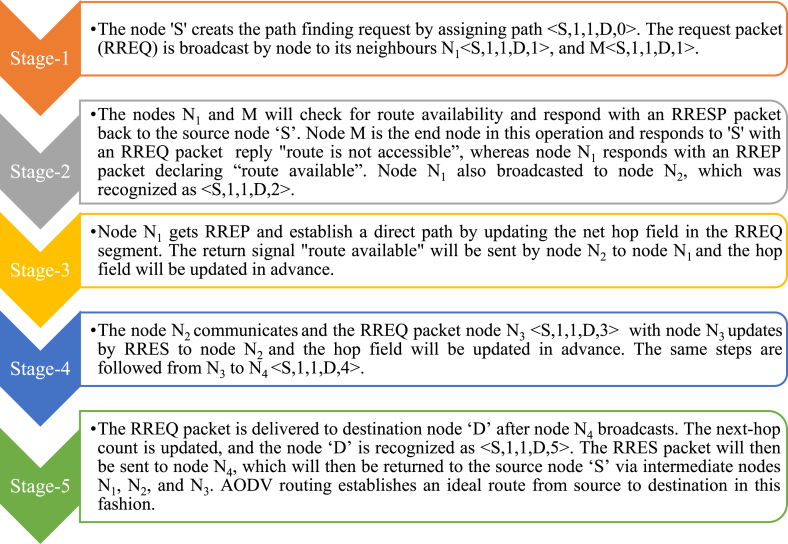
AODV routing steps.

The system knows the source and destination node IP addresses. The following fields are used to route in AODV: RREQ (IP destination, the sequence number of destination, IP source, the sequence number of source, and hop count). The goal of AODV routing [48] is to process data packets by recognizing, realizing, and sustaining the best route among communicating nodes. The data packets are delivered from source to destination to identify, realize, and continue the optimal track between source and destination. The source node ‘S’ communicates to the destination node ‘D’. The behavior can be understood with the AODV capabilities and the steps given in Figure 6. The approach is applicable for network control and routing in WSN [49]. WSN and MANET are emerging networks used for multi-hop communications in mobile networks and industrial contexts such as NCS. Industrial ad-hoc networks are built on the time-variant network topology. AODV routing supports bidirectional connections [50] and routing algorithms that can be expanded to function in the presence of unidirectional channels in the tradeoff of larger overhead.

## 5 Results & discussions

In contrast to data networks, the architecture of the NCS network faces difficulties with the small but time-sensitive data packets. The NCS as a whole can become unstable because to packet delays and dropouts. The packets must include details about frequent small packets, constrained packet delays, and high-quality packet delivery. In addition, maintaining a stable NCS presents some difficulties, including precisely calculating and maintaining acceptable packet drop rates, compensating for incomplete or delayed sensor data arrival, implementing the best real-time controller task scheduler, compensating for irregular/inappropriate sampling, and dynamically adjusting the sampling interval and average packet delay. These factors must be taken into account when the Controller determines the update for the plant based on the received state, as the transmission delay to the plant may have an impact on subsequent states. Network Simulator-2 (NS-2) is the most widely used network simulator tool for Ad-hoc network simulation.

The MANET simulation model is built on the WLAN Zigbee standard, in which 150 nodes are configured. The objective of choosing the standards is that they can be applied in NCS modeling which can be used for Ultra-Wide Band (UWB). The Zigbee frequency range is generally 2.4 GHz worldwide. This does not, however, imply that 2.4 GHz is always supported. Mesh topology is used to configure the Zigbee nodes in MANET. The channel is multi-hop and bidirectional. ZigBee can transfer data over distances of up to 50 m and has a maximum data transfer rate of 250 kbps. The latest embedded devices can transfer at lower data rates of 20 and 40 Kbit/s. Internet protocol version 6 (IPv6) is a multi-hop routing protocol used for low-power and loss networks to connect Zigbee nodes. It is possible to organize non-interfere MANET channels for each ZigBee channel using existing sensing methods for ZigBee channel discovery. WSN sensor nodes employ ZigBee at the data link Open Systems Interconnection (OSI) layer. The sensor network is deployed in the area (900 m × 900 m) to see the parameters in real time and dynamically see the parameters of the network. The network is being designed keeping in mind that the nodes can redirect traffic based on the new network structure, the network does not need a standard infrastructure. Mesh architecture and ad-hoc routing features give improved stability in varying situations or single node failure. The simulation is carried out with the following simulation environment as listed in Table 1. When AODV is set up in NS2 as a routing protocol in the TCL script with the command $ns node-config-adhoc. The pointer changes to the ‘start’ and from there, it moves to the AODV protocol's command function. The source locates two timers in the ‘start’ parameters using the command function: Currently, the node may see scheduler and instance because the pointer is in the AODV to send Hello. The packets will be scheduled by the function schedule for the target.

**Table 1:** Simulation environment.

Description/Parameter	Value
Simulation Area	900 m × 900 m
Nodes	150
Packet Size	1024 Bytes
Control packet size	256 Bytes
Simulation time	320 s
MAC-Multichannel	IEEE-802.15.4
Traffic source	UDP(CBR)
Communication Range	400 m
Model for mobility	Bidirectional and random
Pause time	150 s
Node speed	10 m/s

**Figure 7:**
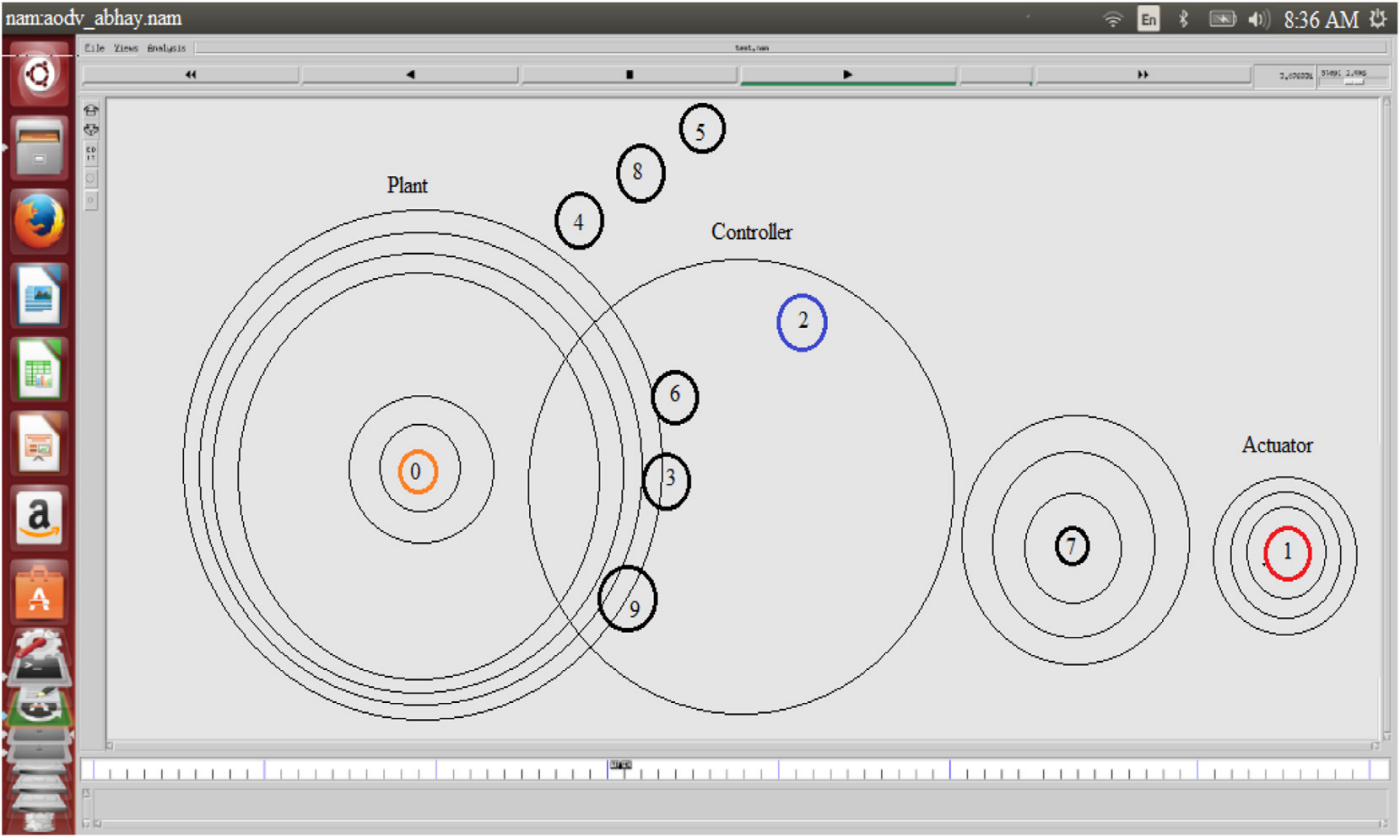
NAM-NS2 simulator view for NCS.

**Figure 8:**
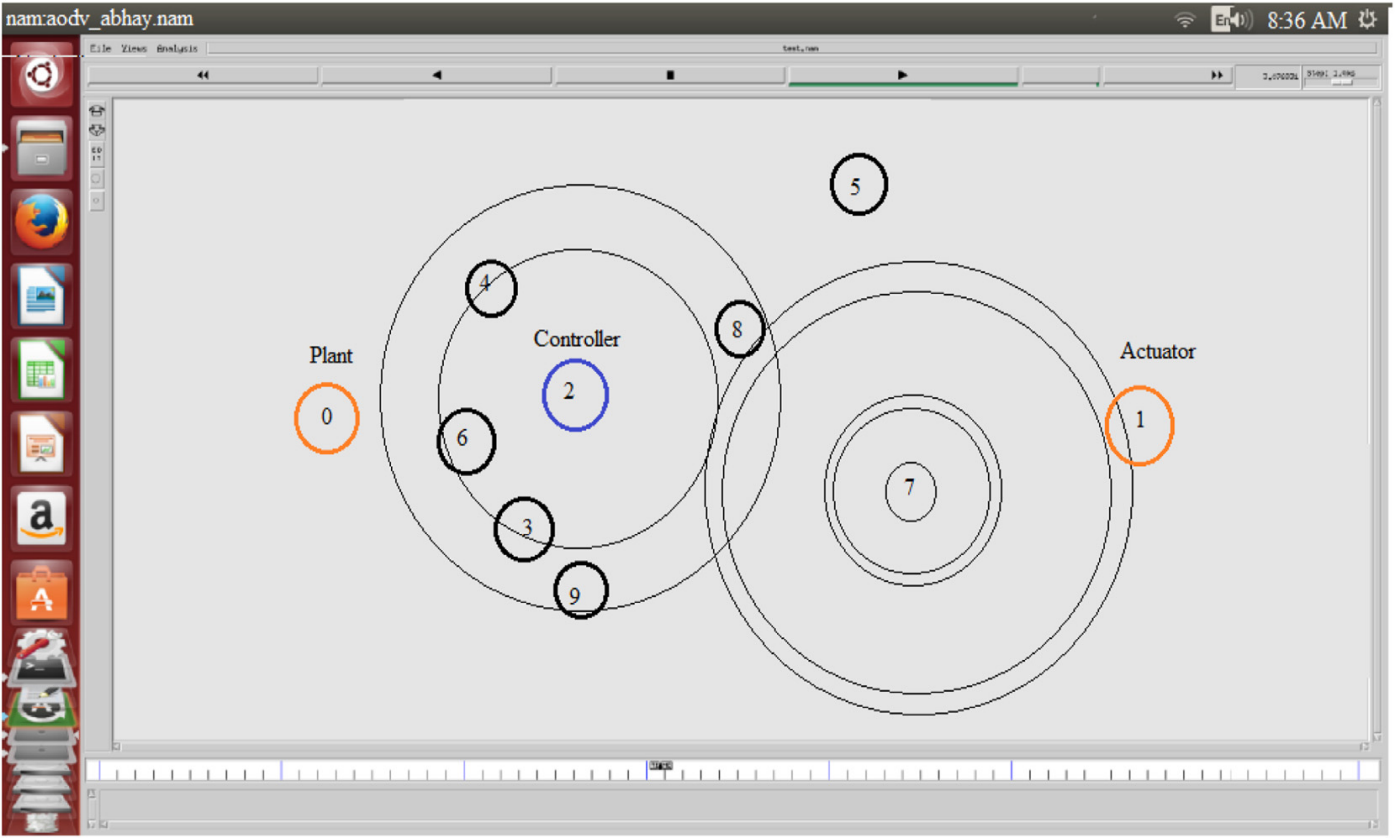
NS2 simulator view for NCS for data control from source to destination.

**Figure 9:**
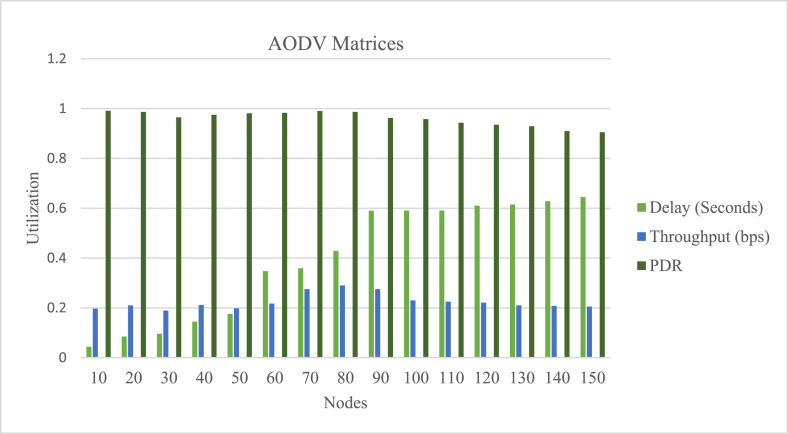
MANET nodes in AODV, variations, and parameters (delay, PDR, and throughput).

**Figure 10:**
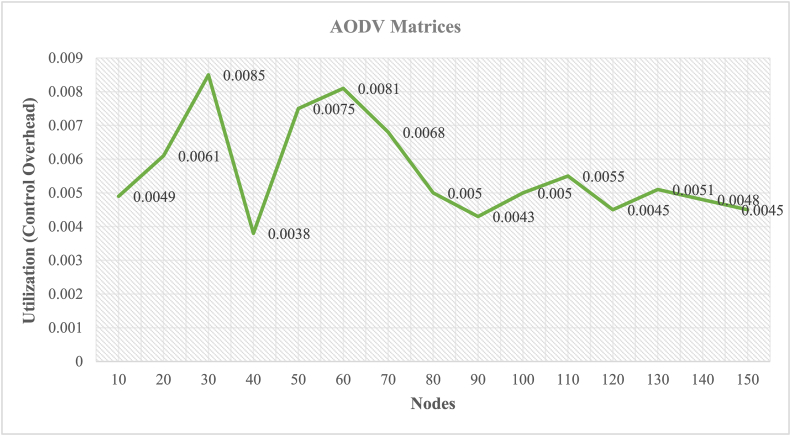
MANET nodes in AODV, control overhead parameter.

The NS2 was chosen to implement NCS on MANETs as a result. The behavior of the node is simulated in NS2 for real-time communication using the Object Tool Command Language (OTCL). The NS2 simulation software simulates AODV routing for various cluster nodes. The work is carried out to implement the NCS on MANETs. The simulation results are saved as NAM and. tr files. Figures 7 and 8 illustrate the NAM snaps. In case-1 the controller node-2 can communicate with the nodes (6, 3, 9), (4, 5, 8), and 7 using AODV. In case-2 the controller node-2 can communicate with the nodes (6, 3, 9 4, 8), 5, and 7 using AODV. The graphs for the throughput of NCS are extracted from the findings obtained from the. tr files. Different performance indices, such as end-to-end delay, throughput, packet delivery ratio, and control overhead, are used to evaluate the AODV-NCS system's performance. The values of the parameters are listed in Table 2. The graph that corresponds to the analysis is shown in Figures 9 and 10. As the number of nodes grows, the delay is expected to increase. The packet delivery ratio is good when control overhead and throughput is optimal.

**Table 2:** Performance parameters for AODV.

	Delay (s)	Throughput (bps)	Control overhead	PDR
10	0.044	0.197	0.0049	0.991
20	0.085	0.210	0.0061	0.987
30	0.096	0.189	0.0085	0.965
40	0.145	0.212	0.0038	0.975
50	0.176	0.198	0.0075	0.981
60	0.348	0.217	0.0081	0.983
70	0.359	0.275	0.0068	0.990
80	0.429	0.290	0.0050	0.987
90	0.590	0.275	0.0043	0.962
100	0.591	0.230	0.0050	0.958
110	0.591	0.225	0.0055	0.943
120	0.610	0.221	0.0045	0.935
130	0.615	0.210	0.0051	0.929
140	0.628	0.208	0.0048	0.910
150	0.645	0.205	0.0045	0.905

It has been observed that the end-to-end latency is increasing and varying from 0.044 to 0.645 s as the cluster nodes are changing from (N = 10, 20,…,150). The reason for the increased latency is obvious it will increase as the nodes will increase. The throughput is changing from 0.197 bps to 0.290 bps with different node clusters. It is dynamic with different cluster nodes. The control overhead is also dynamic but optimal. The value of the control overhead is varying from 0.0038 to 0.0081. The nodes in the AODV routing remain completely silent as long as there are no packets to broadcast. As route requests are received and the connection is formed, the source node must flood the network. After receiving the route request, the destination sends the route response to the source, which is based on one of the routes discovered during the route request phase. In networks where the vast majority of nodes have nothing to send and connections entail more than a few packets, this strategy plays out in terms of lowering total routing overhead. PDR behaves similarly near maximum utilization regardless of the number of nodes, but above 80 nodes, its behavior changes. The likelihood of collision increases as the cluster size grows and the number of nodes in the scenario grows, resulting in a lower PDR after 80 nodes. The value of PDR is varying from 0.991 to 0.905 for (N = 10, 20,…, 150).

The data presented in the table is used to create and validate statistical models. The procedure entails performing training and testing operations on data collected for various characteristics such as latency, throughput, PDR, and control overhead. The comprehensive data is separated into separate training and testing. Because even a little amount of biasing can lead to incorrect model estimates and performance evaluation, the testing method at this stage ensures that no biasing is possible in the final model estimation. The entire procedure entails preprocessing parameters, dividing data into training and test groups, applying mathematical processing, selecting appropriate models, and assessing system performance. The technique of modeling the linear relationship between a target node (dependent variable) and the predictor is followed using the multiple linear regression (independent variable) techniques. The ML regression model [50] is based on the assumption that errors are distributed uniformly with constant variance and zero means. The direct estimated value of **R**^**2**^ for 150 estimators is 0.9516, 0.9715, 0.9253, and 0.9810 for the delay, throughput, PDR, and control overhead respectively. It is observed that the PDR declines with the larger number of nodes and the delay is increasing with the size of cluster nodes.

## 6 Conclusions

A new class of control system called a wireless networked control system is created when wireless routing is employed as a communication medium in a control system. The MANET Zigbee nodes not only carry out the same data transmission and reception functions as the existing host, but they also act as routers. The mobile nodes provide dynamic routing in route settings and multipathing to neighboring nodes. The results estimate the system behavior with different indices and nodes. It is analyzed that the delay is increasing from 0.044 s to 0.645 s with nodes (N = 10, 20…150). The throughput is varying 0.197 bps to 0.290 with different node clusters. The control overhead value is changing from 0.0049 to 0.0085 with different cluster configurations. The maximum throughput is achieved in most cases. In the future, we are planning to embed the concept of network security with the cryptographic hardware chip that can support more nodes in NCS. The network is not limited to 150 nodes, depending on the distance between the nodes, topology configuration, and control overhead. The novelty of the work is to specify the traffic generation rate in the simulation script and alter it based on the user requirements makes the work distinctive. Additionally, the special feature of this work is that it enables the acceptance of user-defined data in the application layer and the transmission of that data to the transport layer agent as the payload of simulation packets. The NS2 simulation work provides the estimation of the parameters for different cluster sizes that will help the hardware design engineers to pre-estimate and configure nodes in the scalable network so that optimal embedded hardware chip solutions can be intended for specific NCS controlled by WSN and MANET. Fogify emulator [51] is a suite of tools for modeling complicated topologies with diverse resources, network capabilities, and QoS criteria. The modeled configuration and services can then be deployed utilizing common containerized infrastructure and local environments. In the future, we intend to analyze network performance on the Fogify emulator for a large number of nodes and network size.

## Declarations

### Author contribution statement

Abhay Bhatia, M. Tech: Conceived and designed the experiments; Performed the experiments; Analyzed and interpreted the data; Wrote the paper.

Anil Kumar, Ph. D: Conceived and designed the experiments; Wrote the paper.

Arpit Jain, Ph. D: Analyzed and interpreted the data; Wrote the paper.

Adesh Kumar: Performed the experiments; Wrote the paper.

Chaman Verma, M. Tech; Zoltan Illes, Ph. D; Ioan Aschilean, Ph. D; Maria Simona Raboaca: Contributed reagents, materials, analysis tools or data; Wrote the paper.

### Funding statement

This work was supported by Ministerul Cercetării, Inovării şi Digitalizării (PN 19 11). The work of Chaman Verma and Zoltan Illes was supported by the faculty of informatics. Eotvos Lorand University, Budapest, Hungry.

### Data availability statement

Data will be made available on request.

### Declaration of interest's statement

The authors declare no conflict of interest.

### Additional information

No additional information is available for this paper.
